# Distributed Architecture for Unmanned Vehicle Services †

**DOI:** 10.3390/s21041477

**Published:** 2021-02-20

**Authors:** João Ramos, Roberto Ribeiro, David Safadinho, João Barroso, Carlos Rabadão, António Pereira

**Affiliations:** 1 School of Technology and Management, Computer Science and Communication Research Centre, Polytechnic Institute of Leiria, Campus 2, Morro do Lena-Alto do Vieiro, Apartado 4163, 2411-901 Leiria, Portugal; jr.joaoramos@outlook.com (J.R.); eng.rob.ribeiro@gmail.com (R.R.); davidsafadinho.12@gmail.com (D.S.); carlos.rabadao@ipleiria.pt (C.R.); 2 INESC TEC and University of Trás-os-Montes e Alto Douro, Quinta de Prados, 5001-801 Vila Real, Portugal; jbarroso@utad.pt; 3 INOV INESC Inovação, Institute of New Technologies, Leiria Office, Campus 2, Morro do Lena-Alto do Vieiro, Apartado 4163, 2411-901 Leiria, Portugal

**Keywords:** centralized services, cloud platform, cloud services, distributed architecture, Internet of Unmanned Vehicles, unmanned vehicles

## Abstract

The demand for online services is increasing. Services that would require a long time to understand, use and master are becoming as transparent as possible to the users, that tend to focus only on the final goals. Combined with the advantages of the unmanned vehicles (UV), from the unmanned factor to the reduced size and costs, we found an opportunity to bring to users a wide variety of services supported by UV, through the Internet of Unmanned Vehicles (IoUV). Current solutions were analyzed and we discussed scalability and genericity as the principal concerns. Then, we proposed a solution that combines several services and UVs, available from anywhere at any time, from a cloud platform. The solution considers a cloud distributed architecture, composed by users, services, vehicles and a platform, interconnected through the Internet. Each vehicle provides to the platform an abstract and generic interface for the essential commands. Therefore, this modular design makes easier the creation of new services and the reuse of the different vehicles. To confirm the feasibility of the solution we implemented a prototype considering a cloud-hosted platform and the integration of custom-built small-sized cars, a custom-built quadcopter, and a commercial Vertical Take-Off and Landing (VTOL) aircraft. To validate the prototype and the vehicles’ remote control, we created several services accessible via a web browser and controlled through a computer keyboard. We tested the solution in a local network, remote networks and mobile networks (i.e., 3G and Long-Term Evolution (LTE)) and proved the benefits of decentralizing the communications into multiple point-to-point links for the remote control. Consequently, the solution can provide scalable UV-based services, with low technical effort, for anyone at anytime and anywhere.

## 1. Introduction

The evolution of the technology is raising new challenges and simultaneously answering them. The services as people know are changing, becoming closer and easier to access than ever [1]. For a simple task like buying groceries [2], we had to walk or drive to a local shop and buy the products in loco. Now, there are shops available online at a distance of a click. In just a couple of hours or days, the groceries are left at people’s door. This is one example of how services can be improved to be more transparent and easily accessible.

Meanwhile, the world is getting more connected. The evolution of communication technologies, such as the Internet, or mobile networks like Long-Term Evolution (LTE) and more recently 5G, is helping to connect people from any part of world and let them contact each other in an instant [3,4]. This led companies to start to reorganize their structure and replan their business logic, to reach as many people as possible [5].

Relative to new communication protocols, each device will potentially have some sort of connection to somewhere. This concept of devices interconnection is commonly known as Internet of Things (IoT) [6]. Examples of that evolution are in almost every new house: Closed-Circuit Television (CCTV) cameras, smart lights, smart blinds, and doorbells. Even cars are becoming always connected to the cloud to provide owners with information such as their parking location and even perform automated tasks—Connected and Automated Vehicles (CAV) [7]. This globalization, combined with the small scale of the new devices, is also known as smart dust, that means that we are surrounded by many small particles with embedded intelligence [8].

At the same time, vehicles are also facing a huge transformation. The airplanes have automations for taking off, cruising and landing. It will not be a surprise to see soon airplanes and helicopters transporting freight and people without a pilot among the crew. Thanks to the Advanced Driver Assistance Systems (ADAS), many cars built nowadays have autonomy to drive for themselves for a certain period of time. Therefore, it will not be a surprise, as well, to see a car without a driver, rather just with passengers. This can bring us to the following questions: what about a car that is simply delivering a parcel? What about an airplane that is just dropping water to combat a wildfire in the forest? Is it possible to reinvent the meaning of using a car, a boat, or an aircraft in such situations?

Trying to answer these questions will lead to the field of unmanned vehicles (UVs). Vehicles without a pilot or any human on board may become smaller, lighter, more cost efficient, and easier to manage than the regular ones. Instead of using a truck to deliver the parcels, it is possible to rely on a drone. In fact, this alternative is already being tested and done by companies such as Amazon and DHL [9,10]. Other applications of UVs include surveillance [11], ambient variables measuring (e.g., temperature, air humidity) or even just recreation [12,13]. Difficult and/or dangerous tasks that involve some kind of risk to the humans onboard (e.g., aerial electricity lines inspections, in which helicopters may be requested [14]) can also be slightly improved through UVs.

The growing of UV applications faces challenges, as well [15]. Most people may not have access to a drone or a remote-controlled (RC) boat or will not be motivated to invest money on a device that they will likely use just a few times. Even if they have that possibility, they may not have the knowledge to control them to fulfill some necessities. We do not expect everyone to manually control a drone and deliver a parcel by themselves. Then, this is a good opportunity to provide transparent services to people, in which they can simply focus on their objectives and forget about the logic and operation behind them. The resources allocation will be optimized, both human and material, as the vehicles may be shared by the different services.

The combination of unmanned aerial vehicles (UAV) and Internet services is already proposed as the concept of Internet of UAV (IoUAV) [16,17,18]. The Internet and drones in specific are also related in [19,20] as Internet of Drones (IoD). Finally, all the vehicles may also communicate between them through the Internet, which is described as the Internet of Vehicles (IoV) [21]. To the best of our knowledge, there are no mentions about a concept that interconnects different UV types (i.e., terrestrial, aquatic, aerial). Then, with our solution, we introduce the definition of Internet of Unmanned Vehicles (IoUV), a concept that promotes the symbiosis of different UV types and leads to the creation of many and flexible services, independent of the travel environment. Challenges arise as services become an interest, such as how can they be implemented and centralized to become easily accessible and deliverable to anyone, instead of providing fragmented services, available separately. As an extension of a previous publication, this paper responds to these challenges, by answering to the following research question.

**Research question:** How to create a scalable solution to provide UV-based services, accessible anywhere, anytime, and for anyone?

This work provides a solution for centralized services, using UVs, to solve common problems. In order to answer the research question, the goals of this work are the analysis of the solution’s requirements and the definition and proposal of an appropriate and distributed architecture. Moreover, this work intends to define the methods to include and integrate several vehicle types that can collaborate to provide new services for different application fields. Instead of creating a fragmented set of services, they can be combined into a single solution that supports a more intuitive and transparent manner of delivering them. Thus, the contributions of this work are the following:

- Centralized services, combined in a single solution;
- Promotion of new generation UV based services for any type of user, through a robust and highly available cloud platform;
- Make transparent the usage of UV via high-level methods with embedded safety features;
- Promote the integration and collaboration of different vehicle types to work together;
- Creation of new UV-based services with low effort which increases the efficiency of production for developers and companies that want to release new services;
- Proposal of a distributed architecture through multiple point-to-point connections, that promotes the expansion and scalability of any system;
- Proposal of the novel concept of IoUV, which integrates sensing and actuation in all kinds of unmanned vehicles, either autonomous or controlled remotely.

The structure of the article is as follows. In the Section 2, the UV platforms are reviewed, considering how they are implemented, how they provide the services and how can they scale on the addition of new services and vehicles. Then, considering the existing solutions, a new approach is proposed in the Section 3. We present and discuss the solution’s requirements and architecture and introduce two examples of services to explain and enhance the strengths of the platform in such situations. Section 4 describes the prototype implemented according to the proposed solution, considering the cloud platform and several UVs from three different types. For this work, the available services comprise the manual control of each UV, from a tablet or a computer. In Section 5, the prototype was subject to functional tests for its consequent validation. Section 6 presents the evaluation the prototype, regarding the performance of the communication, and discusses the results in order to validate the feasibility and employment of the proposed solution in our society. Based on the learned lessons, we also present recommendations to get the best outcomes with the proposed solution. Finally, Section 7 presents the conclusions and discusses the future direction of this research.

## 2. Unmanned Vehicles Platforms

The implementation of UV platforms requires the definition of appropriate architectures. This section presents different solutions for the communication between users and vehicles. In the first subsection we present communication architectures for user–vehicle interactions, whilst the last subsection presents the related work about UV platforms research: how they work, which vehicles they support and how they scale. Although this work intends to create a generic solution for the different types of UVs, the first subsection focus specially on UAV communication architectures, since the latest developments and research are mostly related to the IoD concept [19,22].

### 2.1. Communication Architectures

Users should be able to send information to vehicles and vice versa. This is often achieved through Ground Control Stations (GCS). This infrastructure, usually installed by the users in their own equipment through software and hardware modules, communicates with the vehicles to send commands and receive data.

The GCS-UV connection is possible through different architectures. In [23] the authors present four approaches: one centralized and the others decentralized. In the first one, the GCS is responsible for the communication to all vehicles. In the remaining architectures, the vehicles have autonomy to communicate with each other. The first example of a decentralized architecture includes a single vehicle communicating with the GCS. Then, this vehicle acts as a relay point for the other UVs. The next example is similar, existing more than one vehicle acting as relay for the others. Finally, the fourth example shows cooperation between the relay vehicles, reducing the GCS dependency during the missions. The decentralization contributes for mitigating communication bottlenecks and single points of failure.

The definition of an architecture is preceded by the definition of protocols that ensure seamless communication and integration for both the users and the vehicles. In this sequence, a framework layer entitled Robot Operating System (ROS) was created [24]. This framework sits on top of the UV’s operating system and contributes for their integration. The conception of ROS made creation of autonomous, semiautonomous and manually controlled vehicular systems easier. Later, an open-source communication protocol named Micro Aerial Vehicle Link (MAVLink) was created [25]. This protocol includes a set of bidirectional communication messages, that can be extended depending on specific needs [26] and allows the control of multiple vehicle types. This paper goes through the same principles: define and create protocols to control and monitor UVs [27]. One of the downsides of MAVLink is that users and developers will find extensive and complex documentation and procedures to use this protocol. This led to the emergence of UV cloud platforms, as users should have straight access to the applications, without the need to understand the protocol implementation details. FlytBase and FlytNow are examples of UAV cloud platforms, that provide users with ready-to-use services, based on MAVLink messages and LTE mobile communication networks.

The communication between users and UVs can be established in different manners. Users should have easy-to-use, intuitive, and effortless methods for this step. Through the GCS, the connection links can be ensured via radio or via the TCP/IP protocol. The first approach uses one dedicated transceiver and one dedicated receiver in the user and vehicle sides, respectively. The TCP/IP protocol works as long as users and vehicles are connected to the same network. Hence, the same network can be shared by multiple users and interfaces, for example through Wi-Fi.

**Figure 1:**
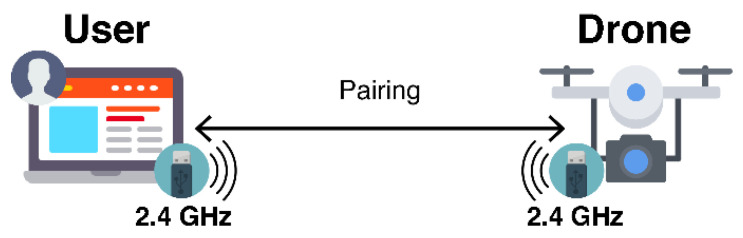
Pairing between the user and the drone through 2.4 GHz radio.

**Figure 2:**
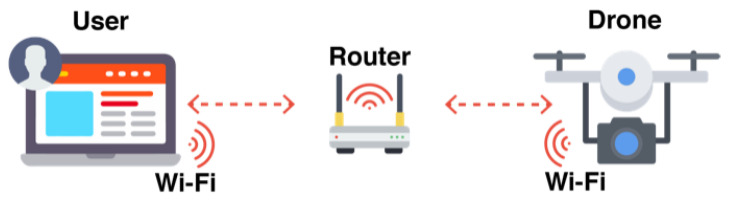
Local network architecture representing a user communicating with a drone via a Wi-Fi router.

In Figure 1 the method for establishing connection through a 2.4 GHz radio link between the user’s controller and the transceiver installed in the vehicle is represented. First the user should complete the instructions provided by the transceiver manufacturer to pair the radio modules. On each communication link, multiple channels are available to transmit different commands (e.g., throttle, rotation). In Figure 2 the TCP/IP network procedure is represented. It is more complex than the radio fashion, as users need to understand about network management. The procedure demands that the vehicle connects to the GCS. Then, the users need to configure their Internet Protocol (IP) address in the vehicle to establish the connection.

The network method allows users and vehicles to be in different locations and communicate remotely, mainly via the Internet, unlike 2.4 GHz radio links that have a restricted range, also limited when the line of sight is blocked. Although, the configuration of the networks routers in both sides is highly complex. Users must make advanced configurations and share their personal devices’ information (e.g., IP address) to the vehicle. Thus, this work proposes a new solution to make the remote communication between the users and UVs as transparent, intuitive and effortless as possible, simultaneously providing high-level methods for developers to integrate different vehicle types and services.

### 2.2. Platforms as Services

After discussing how typical vehicle communications work, we searched for platforms that offer services using UVs. Therefore, in this subsection we present the platforms that partially answer the research question.

FlytBase Cloud [28] is an enterprise solution that claims to be a “Drone-API (Application Programming Interface) as a Service”. The basic principle is to provide endpoints for accessing drones in real-time and integrating them with 3rd party applications. They have compatibility with Python and C++ languages, as well as with the Representational State Transfer (REST) and WebSockets protocols. The documentation separates the interfaces as Onboard APIs (Python and C++) and Remote APIs (REST and WebSockets). Following these two approaches, it is possible to directly control the vehicles remotely or create new applications inside them. The endpoints in the FlytBase APIs are similar to the ones found in the MAVLink protocol, including arming/disarming the motors, navigating to a waypoint, defining the vehicle’s speed in each axis, taking off and landing. Although, it may be prone to errors either from developers or users, as the documentation is wide-open and still focused on the drones’ specificities, like their flight modes (e.g., guided, offboard, manual). Furthermore, FlytBase is only compatible with drones, which means that there is no possibility for cooperation between different UV types (i.e., aquatic, terrestrial).

The DroneMap Planner [20] presents a solution for UAV collaboration through the cloud and supports MAVLink compatible vehicles. The main purpose is to open the possibility of creating multiple applications using drones and the Internet, once more featuring IoD. Providing Web Services and WebSockets for the different drones’ actions, the authors created different services including, for instance, object tracking. To use this service, users request the cloud to follow a target, and the platform allocates an available drone. After the user starts the tracking and while they send specific GPS locations, the cloud manages the drone’s control. Hence, the user does not need to know how to control a drone. The actual commands and actions are responsibility of the cloud, that can accommodate different application logics. For the cloud–drone communication, they use Web Services and WebSockets. The cloud has the intelligence to decide what to do next and send the corresponding command to the vehicle, that will finally execute it. Although the vehicles do not require any changes to be used by other services, the cloud may suffer from bottleneck as all actions and processing are performed there.

DroneMap Planner also includes an API written in Java that developers can use to build new applications. Via the server’s IP Address, users can connect to an available UAV and send specific commands like takeoff or land. However, releasing the direct access to the drones’ functionalities, without considering safety validations, leaves gaps for unattended behavior and puts at risk the safety of the users, the vehicles, and the surrounding environments.

In [29], the authors created a solution for multiple UAVs control through a single GCS. The users access the GCS through the Internet to fetch the correspondent user interface. The purpose of the system is to monitor specific areas to search and rescue in disaster situations. Thus, users may select, through the user interfaces, the areas that they intend to analyze, while they monitor in real time the mission’s progress. The users communicate directly to the GCS for the mission planning, control and analysis, whilst the GCS is responsible for the communication and articulation between the vehicles. This solution does not present ways for developers to create new applications and is restricted to UAV usage only.

In [30], the authors brought the concept of Web of Things for the integration of UAVs in the cloud. To achieve this goal, the authors defined the UAVs as the Cloud Infrastructures themselves. Each vehicle represents one back-end server and waits for REST connections. The users access a web interface through another server, hosted in the cloud, that displays a list of all the connected drones. From the same web interface, users can request the UAV to capture images or return home, for example. By providing powerful REST endpoints directly to the users (e.g., manual takeoff or landing), each vehicle is exposed on the Internet and may be vulnerable to undue requests and actions. To prove the solution, a prototype was created based on an Arduino microcomputer, without flying capabilities, but equipped with different sensors, whose sensorial information was available through a group of web services.

In [31], the authors present a cooperative solution with an Unmanned Ground Vehicle (UGV) and a UAV. The purpose of the system is to perform autonomous power lines inspection, by using both vehicle types to enhance the efficiency and efficacy of the operations. Both vehicles are connected through Wi-Fi to a base station and share their location in real-time. The vehicles follow each other and cooperate to find obstacles during the missions. As both are analyzing for the possible dangers, the system runs more safely than if they were performing solo. Despite this cooperative architecture between two different vehicle types, there is no mention about the integration of new applications or vehicle types.

The IoT concept brings more opportunities to the development of services. Following the tendency, the authors of [32] proposed an IoT surveillance system, based on a decentralized architecture, which includes three different layers: human, hidden and environmental. The first layer includes the actions and control of the processes performed by the human operators. The hidden layer includes all the processing associated with the data gathered from the environmental layer. This last layer includes all the smart devices responsible for the in-site surveillance and sensing. The devices, also interpreted as machines, may include UGVs, for example. Even though the architecture communication follows the order of the human → hidden → environmental layers, there is also a communication link from the humans directly to the machines, named H2M. This work is specialized in a single application area and proves to be efficient when using multiple UGVs for the surveillance of a building. Although the paper includes a generic architecture for UGVs usage, it does not present alternatives for the integration of different applications or vehicle types.

In [33], the authors present a collaborative solution, that includes ground robots and UAVs for autonomous mapping in search and rescue missions. Both ground robots and UAV connect via Wi-Fi to a network router and communicate through the ROS protocol. Whenever a relevant event is identified by the system (e.g., a person is detected), the rescuers receive a notification. The UAV also provides the rescuers with a 2.5-dimensional map. The authors conclude that with their proof-of-concept integration, the robot–robot and human–robot collaborations have potential. However, there is no mention about the integration of external services.

In Table 1 we summarize the different platforms comparing the supported vehicle types and services, as well as the possibility and complexity of developing new applications. The solutions analyzed are compatible with UAV and/or UGV. When authors present programming interfaces, it is possible to create multiple and different services. Although, they require a medium/high comprehension and knowledge about vehicles’ control logic (e.g., learn the MAVLink protocol, in the case of FlytBase), which increases the development complexity and effort. This is the concern of this work, that proposes compatibility between multiple and different vehicle types, potentiating the creation of new flexible services, whose task is independent of the environment (i.e., ground, water, or air). The proposed solution presents a new approach for creating multiple services through the deployment and decoupling of User, Platform and Vehicle tasks for a seamless and effortless integration. Besides that, when compared to other platforms, that provide low-level API for development, we introduce a higher-level abstraction interface that includes embedded safety mechanisms inside the vehicles. However, this abstraction layer requires an adaptation of the communication module (i.e., hardware or software) for vehicles that do not use common or accessible communication interfaces. Professional UAVs from commercial companies like DJI and Parrot, already provide Software Development Kits (SDK) for vehicle integration. In the following sections we present the new solution, the implementation of the prototype and its validation.

## 3. Solution Requirements and Definition

According to the motivations of this work and the problems identified in the analyzed solutions, this section discusses and proposes a scalable solution to provide services to anyone, from anywhere, at any time. The first subsection analyzes the requirements of the solution, followed by the definition of a distributed architecture and the description of the services’ protocol. The last subsection presents examples of applicable services based on UVs, accessible through the proposed cloud platform.

### 3.1. Requirements

To answer the research question, we first specify the requirements of this solution. They are listed in Table 2 and describe all the mandatory characteristics for the proposed solution. The combination of all these attributes meets the demand for a generic solution capable to provide services, based on UVs, to the most different users, from the naive to the expert, located anywhere in the world. Following these requirements, the proposed solution can integrate virtually any type of vehicle and service, to make them available through transparent, intuitive and effortless interfaces to the users. The communication between the different entities should be, preferably, ensured via the Internet.

### 3.2. Distributed Architecture

**Figure 3:**
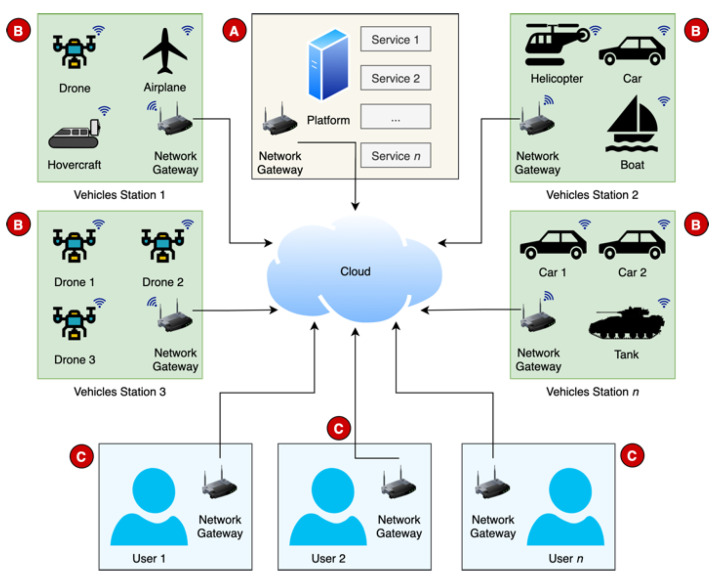
Representation of the architecture, including the three main entities: platform (**A**), vehicle stations (**B**) and users (**C**).

This platform intends to provide users with a variety of services based on UVs. In most services, users need to be distanced from the vehicles, either because of pure convenience or safety measures. It is mandatory to define a well-structured and scalable architecture that expands geographically, in the number of functionalities and in the number of users. For this purpose, we identified and defined the main entities in Figure 3. The whole architecture can be comprehended in three subparts: platform (A), vehicle stations (B) and users (C). The main interface is the cloud platform, accessible via the Internet. In the example there are also four vehicle stations and three users. Each vehicle station can contain different vehicle types, depending on special needs or the environmental characteristics (e.g., if station 2 is near the sea, it may include aquatic UVs). The number of entities in the figure are merely illustrative. The proposal of this scalable solution considers as many users and stations as required, with as many vehicles, from different types, as needed, up to a global scale.

The brain of the whole ecosystem is the platform, identified as (A), that must provide users with the access to the services and contain all the related data. Through the cloud, users should be able to access the list of the available services and select the most suitable for their needs. The platform should also include a back end with administration tools to manage the lists of services, available vehicles, and users, and all the related configurations, such as assigning the specific privileges or services to each user.

The distribution of the vehicles is one of the crucial steps to achieve scalability. Thus, the vehicles should be decoupled from the servers and belong within distinct vehicle stations, identified as (B), based on their type and location. Each station should have access to the cloud to communicate with the platform and consequently send and receive information. Each vehicle is preconfigured to establish a connection to the platform and wait for upcoming tasks.

Users, identified as (C), are another part of the architecture and can have several roles, which can be simultaneous (e.g., service client, platform administrator, service performer, service monitor). They will be spread over the world, in contact with the cloud platform via the Internet. The first step for users with the role of service client is to access the platform and select a specific service (e.g., manual control a vehicle, request a delivery). According to the service needs, the platform will assign the required vehicles (e.g., cars, boats, airplanes, drones, etc.). Each user should be able to access the services through different types of personal devices, mobile or static, and have its own login session, to ensure the processes of authentication and authorization.

As multiple services using different vehicles can be integrated, in the next subsection we propose a protocol to ensure the interconnection of all entities for each and any service.

**Figure 4:**
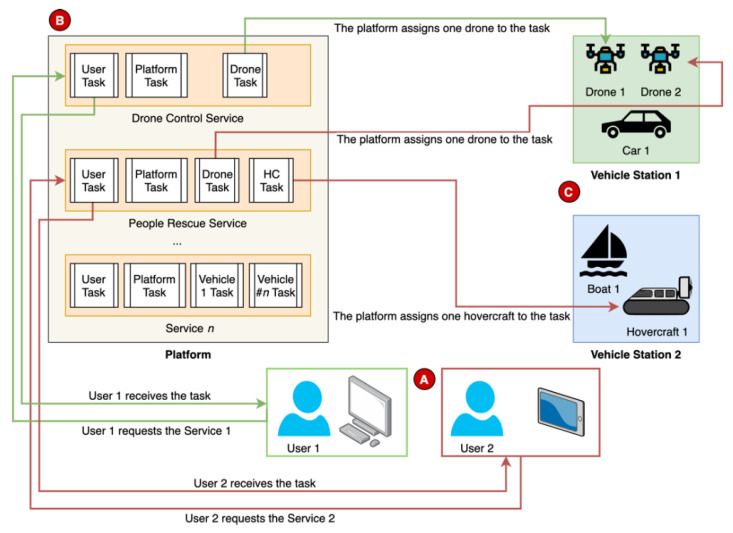
Representation of the tasks’ deployment. Based on the requested services, the platform (**B**) uploads the respective tasks to the users (**A**) and the vehicles (**C**).

The deployment of each task is made by the platform, as illustrated in Figure 4. First, the user (A) requests the platform (B) for a specific service. Afterwards, the platform will assign the vehicles (C) required for that service and send them the corresponding tasks. The user also receives their task, which the platform will run, and all the entities are ready to follow the service protocol.

In the same figure, two different services are exemplified. The User 1 requests a drone control service and receives its interface, which may include a real-time video camera transmission and a keyboard interpreter module to control the vehicle. Simultaneously, the platform assigns one available drone to the service and uploads the task. This task may include the transmission of the camera images, the reception of the commands and the consequent mapping in the motors control. The User 2 requests a service to rescue a person in the sea, which includes the usage of two UVs, one aerial and the other aquatic. In this way, the platform assigns both vehicles and uploads the different tasks to each entity for a seamless cooperation.

### 3.3. Defining New Services

The purpose of the platform is to promote the creation and usage of services that benefit from UVs to solve contemporary problems. Hence, according to the proposed architecture, in this subsection we describe how a service works and the different parts that constitute it. Then, based on the service architecture, we present two examples of service use cases. Finally, we explain the steps to define and integrate new vehicles and services in the cloud platform.

#### 3.3.1. Service Protocol

**Figure 5:**
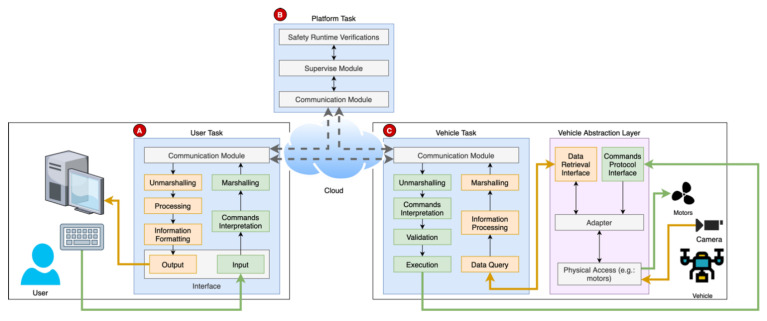
Representation of a complete service protocol. On the left (**A**), the user has the task to control and monitor the service. On the top (**B**), the platform is supervising the service progress and, on the right (**C**), the vehicle is controlled through the abstraction layer.

The services should comply with an identical protocol to ensure a seamless and consistent integration. This protocol includes the different user tasks, the platform, and each vehicle. The user may have an interface specific to the service to issue the commands to the vehicle(s). Then, the vehicle should know how to interpret the received commands and react accordingly. Simultaneously, the platform supervises the whole service and may interact with the user and the vehicles. The protocol is represented in the Figure 5, where the operation of each entity is divided in single tasks.

The user task (**A**) is responsible for presenting the appropriate interface in the users’ device (e.g., smartphone, tablet, laptop). This interface may provide means to interact with the service (e.g., a button to start the service or a map to select a pickup or delivery location). This task is also responsible for the interpretation of the user commands, their processing and transmission to the vehicles and/or the platform.

The vehicle task (**C**) receives the user commands and processes them. Passing along safety validations, the commands are then mapped to actions (e.g., throttle the engines, release the payload, take picture). In the meantime, the vehicle may also transmit flight information to the user and the platform, such as the location and battery status.

The platform task (**B**) supervises the services’ progress, including the user–vehicle interaction. If the user leaves the service or acts inadvertently, the platform task should react and ensure the vehicle’s safety, either by disconnecting the user or instructing the vehicle to discard the received commands.

Whilst the three modules communicate between themselves, the main communication link corresponds to a point-to-point connection between the user and the vehicle. As the vehicles have embedded safety mechanisms in the abstraction layer and even in the vehicle task, the user should communicate directly to the vehicle. In this way, there is no bottleneck in the platform coming from the execution of the service tasks and the solution can grow to any number of vehicles and users, without affecting the performance of the other simultaneous services.

#### 3.3.2. Examples of Services

This section presents two examples of services that can be integrated in the platform. The first one is a synchronous service to control a drone in real-time and the next is an asynchronous service for the rescue in a drowning emergency at sea.

##### Synchronous Service: Manual Control of a Drone

The platform may provide a service to manually control a drone, for either terrestrial surveying or entertainment purposes [34]. In this service, the user controls a drone remotely, using a remote controller or a computer keyboard. According to the input the drone goes up, down, left, and right and rotates to the left and right around its vertical axis. To accomplish this, the following steps should take place:

1. User requests the manual control of a drone in the platform. Optionally, the user may select a location (e.g., a stadium, a mountain, an open area);
2. The platform performs the specific service procedure:
  - Assigns an available drone to the service;
  - Uploads the vehicle-side task to the drone;
  - Sends the user-side task to the user’s device;
  - Runs the platform-side task.
3. The drone waits for the user to establish a connection;
4. The user initiates the connection, according to the downloaded task;
5. A direct communication link is established from the user to the drone;
6. The platform supervises the communication in real-time:
  - If the user compromises the vehicle’s safety, the platform intervenes to protect the vehicle.
7. When the service is finished:
  - Case 1, intentional: the user should inform the platform that it is about to leave;
  - Case 2, unintentional: if the user connection is dropped, the vehicle has autonomy to inform the platform about the situation.

In this use case, the user-side task is designed to establish a connection with the vehicle. Both should use the same protocol and know each other (e.g., HTTP WebSockets through public IP addresses). When the user–vehicle connection is established, the platform stops having a direct participation in the service. It is up to the user and to the vehicle to handle the communication. With this purpose, both vehicle-side and user-side tasks should conform with the message types and content. The user must send the specific messages, depending on the pressed keys, and the vehicle knows how to interpret them and act accordingly. Meanwhile, the platform is monitoring the service in real-time and, in case of danger, it takes an active participation and disconnects the user from the vehicle, for example.

##### Asynchronous Service: Person Rescue at Sea

This is a more complex use case, involving multiple vehicles and asynchronous steps. The main goal of this service is to help rescue a person in a possible case of drowning. Therefore, the service locates the person and delivers rescue equipment such as a floater or a lifesaving jacket. The process should comprise the following steps:

1. The user requests the rescue of a person in a particular sea location, by defining the area of interest in a map provided by the platform;
2. The platform performs the specific service procedure:
  - Assigns the closest aerial vehicle to the service (e.g., drone);
  - Uploads the vehicle-side task to the vehicle;
  - Sends the user-side task for the user;
  - Creates a platform-side task in the platform to be aware of the UAV’s findings and call a lifesaving team to rescue the person.
3. The UAV starts the path, looking for a person in the defined area:
  - The platform waits for a person detection;
  - The user watches in real-time the aerial images via the user application task.
4. When the vehicle detects a person, the platform and the user are notified:
  - The platform starts a second set of tasks, to deliver lifesaving equipment;
  - Assigns the closest compatible vehicle to the service (i.e., aerial or aquatic), that supports payload (e.g., hovercraft or drone);
  - Uploads the vehicle-side task to the vehicle;
  - Creates a platform-task to share the exact location of the person with the rescue team.
5. The new vehicle delivers the equipment in the exact location;
6. The rescue team goes directly to the rescue location and saves the person;
7. Both vehicles go back to their original stations and get ready to new services.

#### 3.3.3. Vehicles and Services Definition

The platform should allow to integrate a virtual infinite number of vehicle types, such as drones, cars, boats, tanks, hovercrafts, and so on. Respectively, each vehicle should be able to accomplish one or more services like goods delivery, surveillance and objects tracking. There is a need to define rules for the integration of each part to guarantee a smooth and flexible inclusion and achieve the genericity we are looking for.

The platform will act as the gateway for all vehicles and services, so an Internet connection is mandatory to achieve worldwide access. Each vehicle must also have an Internet connection to communicate with the platform and the users. The integration of new vehicles starts with the comprehension of the traditional control methods (i.e., proprietary radio remote controller, technology behind the engines, communication protocols). If the vehicle does not include Internet access, external network hardware should be included. Afterwards, the vehicle should have an endpoint (e.g., a software script) to receive executable tasks and follow the services’ protocols.

The main attributes of each vehicle type should be declared and considered. For instance, the drone’s main attributes include flight endurance, maximum range, maximum altitude, maximum payload, type of camera (e.g., none, RGB, thermal, LIDAR, or night vision) and its range. Therefore, every time a vehicle is added in the platform, the values for each of its attribute must be defined.

When a new service is created, it is mandatory to define its requirements. These requirements include the number and types of vehicles that match the description of the task, considering the predefined attributes. Using the two case scenarios presented before as example, the following requirements should apply. For the manual control of a drone in the first scenario, the attributes are not very strict. A drone equipped with a camera is mandatory, the altitude should be limited according to the local laws, and the range may be chosen by the user (e.g., the user can demand high or low range operation). On the contrary, a rescue service implies two vehicles: one aerial and one that supports payload, compatible with the sea environment (i.e., aerial or aquatic). Both vehicles should have autonomy and range to go from the vehicles station to the rescue area. When a life is at stake, time efficiency is critical, which means that, among the available vehicles, the service should choose the fastest. The UAV must be equipped with two cameras to run the person detection algorithms, one with a high resolution RGB sensor and the other thermal. The other vehicle should have sufficient payload capability to deliver the lifesaving equipment.

## 4. Prototype Implementation

**Figure 6:**
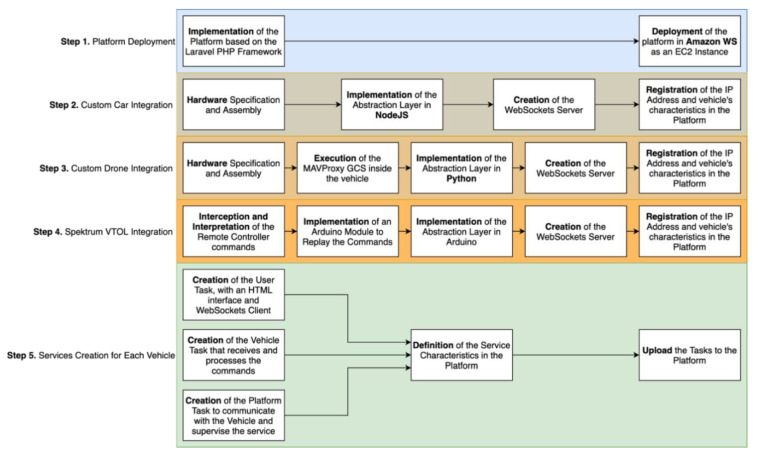
Overview of the steps involved to create the prototype. It includes the platform deployment, the vehicle integration and the service creation.

To validate the solution proposal, we implemented a prototype. An overview of the different prototyping steps is represented in Figure 6. Therefore, Step 1 was the creation of the generic platform that allows to host multiple services. After the platform implementation, Steps 2–4 corresponded to the integration of three different vehicle types. In Step 5, we created a service for the synchronous manual control of each of the vehicles. The same vehicles can be reused in more services created in the future, considering the implementation of the abstraction layers present in each vehicle followed by the WebSockets servers.

### 4.1. Platform Implementation

The developed platform is hosted in a web service so that its role as a cloud platform can be tested in a more realistic scenario. Therefore, the developed platform was deployed to an Amazon Elastic Cloud Computing (EC2) virtual machine available in the Amazon Web Services (AWS) [35]. The EC2 machines are scalability proof through auto-scaling and load-balancing features. If the platform is overloaded with service requests, it is possible to expand and replicate the machines, separating the connected users in multiple instances of the same platform.

The platform is responsible for managing the users, the services and the UVs. After the authentication in the platform through the login page, the user can select the desired service (e.g., control a drone, deliver a package). Then, the platform handles the logic for the specific service and uploads the different tasks to each entity (i.e., users and vehicle(s)).

If the service demands a user interface, it is also the responsibility of the platform to provide it, along with information about its current status. The information displayed to each user depends on its role. If the user is the administrator of the platform, they can add more vehicles or services, while client-users just have access to a service or a group of services.

As the platform is the backbone for the services and vehicles integration, we continue to discuss its implementation in the next sections, with the introduction of services and vehicle implementations.

### 4.2. Vehicle Integration

Before creating the services, it was required to integrate at least one vehicle type. As such, in this section we present the integration of three different vehicle types. For this work, we opted for small-sized and low-cost vehicles. Despite these characteristics, they represent a proof-of-concept of the same integration steps to be applied in any other UV types.

**Figure 7:**
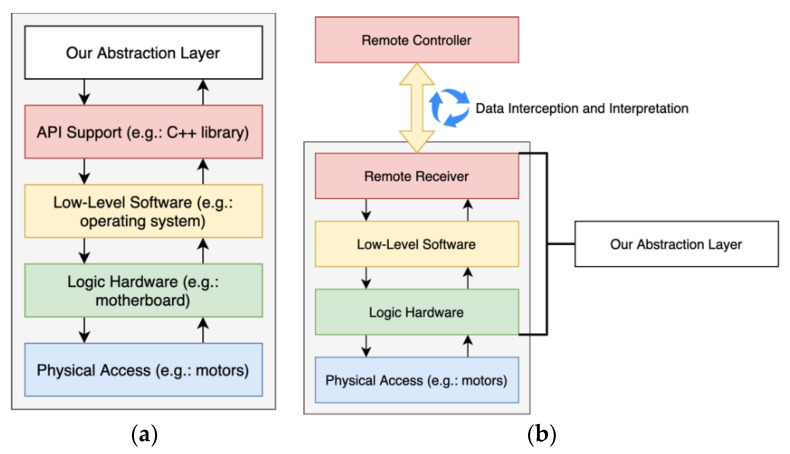
(**a**) Integration of the abstraction layer in vehicles that support development through APIs or SDKs; (**b**) implementation of the abstraction layer on closed-source vehicles that are not planned for development.

The integration process depends on whether they are prepared for development or not. In the case of vehicles with supporting SDKs or APIs, our abstraction layer communicates directly with the APIs, following the manufacturer documentation, as represented in Figure 7a. The abstraction layer can be embedded, without additional hardware. In the case of vehicles that are not designed for development, as represented in Figure 7b, we need to intercept and interpret the data that flows between the remote controller and the UVs’ transceiver. Then, our abstraction layer replaces those modules by replicating the data, either via software or hardware injection. As the worst-case scenario, if it is not possible to replicate all the functionalities, it may be necessary to replace all the logic hardware to match the requirements.

In this prototype we integrated three vehicle types in the platform. Among them there are custom built cars, based on the UK-based Raspberry Pi Foundation’s Raspberry Pi 3 small computer, a custom drone based on the MAVLink protocol and one commercial Vertical Take-Off Landing (VTOL) aircraft based on the Spektrum protocol, named E-flite Mini Convergence VTOL.

#### 4.2.1. Custom Raspberry Pi Cars

**Figure 8:**
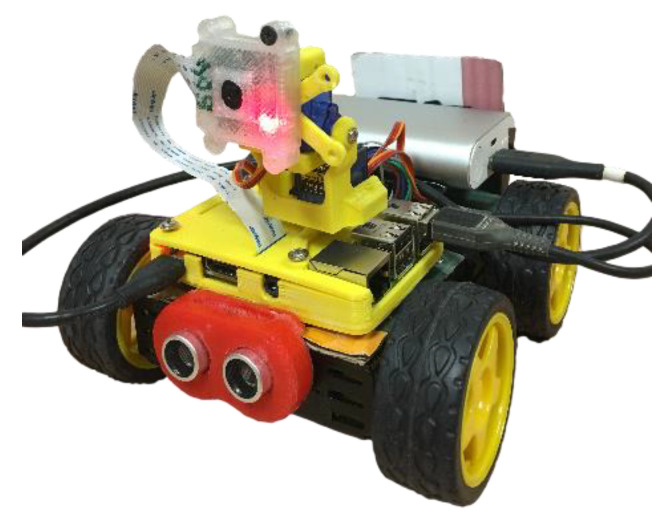
Picture of the developed vehicle included in the prototype as a UV, including the rotating camera and the ultrasonic sensor.

When integrating a vehicle in the platform, the first step is to define the communication method. As explained before, the platform must have an Internet connection, which means that it can communicate with any vehicle with access to the Internet. To build the custom cars we adapted small barebone toy cars using a Raspberry Pi 3 Model B (Figure 8). This small computer has a built-in Wi-Fi module, which allowed to connect the vehicle to the Internet. As the Raspberry Pi runs Linux, we created a Python script to integrate this vehicle type in the platform. This script advertises the characteristics of the cars to the platform and then waits for tasks. In this case, the vehicles can drive forward and backward, while rotating leftward or rightward. One of these vehicles is also equipped with a camera that can rotate along their vertical and horizontal axes, for about 180 degrees, and an ultrasonic sensor on the front for obstacle detection. For this specific car, the service was customized to include the logic to control the camera’s rotation and monitor the status of the ultrasonic sensor.

The motor in each wheel of the car is controlled with the Raspberry Pi 3 and powered through a power bank, mounted on the vehicle. In the end, the Raspberry Pi 3 works as the brain of the car. As input, it is possible to control the motors and the camera arm, and as output it is possible to capture and stream the video to the users.

**Figure 9:**
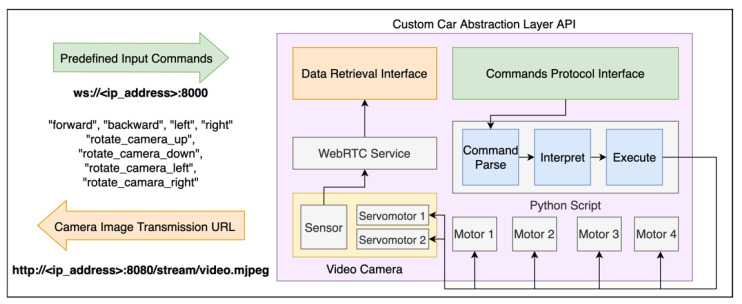
Representation of the abstraction layer built for the custom car. The car waits for the predefined commands and provides a real-time video camera transmission endpoint.

As defined in the architecture, each vehicle requires a customized abstraction layer to make its control and monitoring transparent to the user. The abstraction module for these custom cars is represented in Figure 9. The vehicles must include two distinct interfaces: one for input and one for output. On the left of the image, the available interfaces include the input of predefined commands, through a WebSockets endpoint. Then, these generic commands are processed by the vehicle and converted in the corresponding motors control. As output interface, the cars have the transmission of the camera stream in real-time, through the WebRTC protocol.

Once again, the logic to capture and convert the backhaul video to the WebRTC protocol is done inside the vehicle’s layer and made available as a ready-to-use interface. Using this abstraction layer approach, none of the services require advanced and specific knowledge about how each hardware module works. As fail-safe mechanisms, if the communication with the user becomes instable, the vehicle stops immediately. The same can happen to prevent a crash if the ultrasonic sensor in the front finds an obstacle.

#### 4.2.2. MAVLink Quadcopter

Following specification and implementation of the generic platform, we studied the open-source software MAVLink, which supports the control and monitoring of different UAV types, including quadcopters. To test the MAVLink message protocol, we built a custom quadcopter based a Emlid’s Navio2 shield on top of a Raspberry Pi 3 Model B. This shield based on ArduPilot and ROS works as an autopilot which embeds all the logic required to pilot a quadcopter, including the flight control, telemetry and video transmission, among others.

The default setup for controlling a quadcopter is a radio transceiver, whilst the control data comes from either radio or Wi-Fi to a GCS. Besides, for the scope of this study and to integrate this type of vehicle in the generic platform, we investigated how to control and monitor them through a Wi-Fi connection. Whilst the MAVLink protocol supports TCP/IP connections out-of-the-box, the typical GCSs are limited to the missions’ flight mode, in which the users can only define waypoints for the vehicle to follow. Thus, a custom GCS was required. The first step to configure a GCS is to establish a point-to-point connection with the vehicle. This implies that the vehicle must know how to identify each client (i.e., IP address, network port) and that each client must have advanced knowledge to reproduce the connections. Additionally, providing a direct MAVLink communication to the client would put at risk the vehicle, due to the extension and wide coverage of actions and methods that would become exposed.

**Figure 10:**
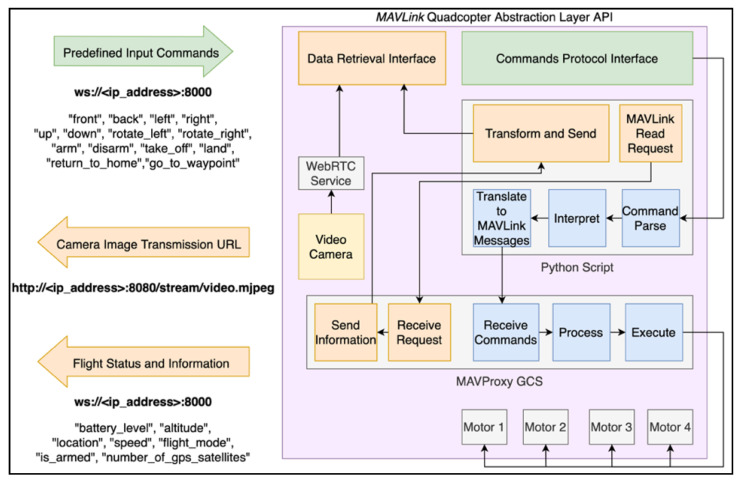
MAVLink based vehicle architecture, to comply with the platform integration. These vehicles receive the commands and send the flight information through a WebSockets connection, while streaming the camera’s video in real-time through the WebRTC protocol.

To create the custom GCS, we designed another solution, represented in the Figure 10. In this solution we run a local GCS inside the vehicle and provide an intuitive interface to the platform, with embedded safety validations and access to a limited set of required actions, instead of every action available in the MAVLink protocol. Then, the platform can connect to the vehicle through WebSockets, as defined in the architecture, and send the tasks according to each service. The abstraction layer has two interfaces, one for input and the other to the output. The input comes from a WebSockets connection and includes the commands presented on the left (e.g., front, back). In the same WebSockets connection, the users can subscribe the messages such as “battery_level” and “altitude”, and consequently receive the updates for these variables. Furthermore, the transmission of the camera stream is also available, through the same *WebRTC* service, supported by Google, used to implement the custom-built cars.

Inside the abstraction layer, the Python script is responsible for transforming the input and output messages. Whenever a user sends a command, it is parsed, interpreted and, if it is valid it is converted to MAVLink messages, according to Table 3. The MAVProxy GCS is the entity that knows how to perform the physical control of the motors. In parallel, the Python script also requests the flight information (e.g., altitude, geolocation) from the MAVProxy, transforms and sends it to the users. Finally, the camera’s video is streamed in real-time, through the WebRTC service.

The Python script interpretation step includes fail-safe mechanisms, whenever the connection between the user and the vehicle faces instability. The vehicle is expecting to receive the commands at a predefined time interval. If there is delay within a certain threshold or the communication drops, the vehicle automatically returns to the take-off location and lands safely.

#### 4.2.3. Spektrum VTOL Airplane

The VTOL airplane integrated in the platform prototype combines the functionalities of quadcopters and airplanes. On one side, drones have the flexibility to take-off and land vertically, in limited spaces. On the other side, the airplanes can glide and achieve high speeds easier. Hence, this VTOL airplane, also known as hybrid VTOL, combines these benefits, by switching from one type to another during the flight. During take-off, the propellers are turned up to resemble a drone. Then, it is possible to switch to the airplane mode, in which the motors turn 90 degrees by using the attached servomotors. While the drones change the rotors’ speed to move in different directions, while in airplane mode, this VTOL can take advantage of the full motors thrust to speed up, controlling the altitude and rotation with a set of moving flaps.

**Figure 11:**
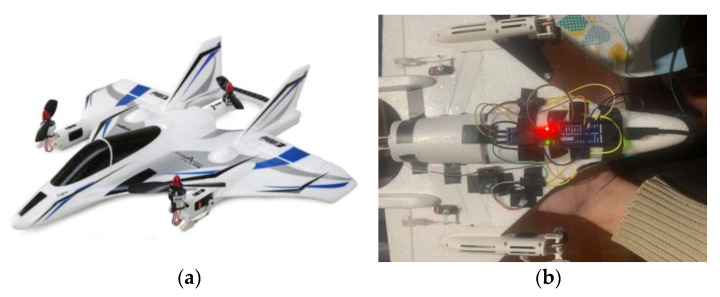
Representation of the Spektrum VTOL Airplane: (**a**) The original E-flite Mini Convergence VTOL; (**b**) the attached hardware to get Wi-Fi onboard and consequently an Internet connection.

To include the VTOL airplane in the platform, we explored a prebuilt kit named E-flite Mini Convergence VTOL, represented in Figure 11. This aircraft has two motors on the front, attached to servomotors. They can either face up or forward. In the rear center, it has a fixed motor. Furthermore, it has one flap on each side to control the rotation and altitude while on airplane mode.

This VTOL is equipped with a proprietary controller. The instructions guide users to use a receiver compatible with the Spektrum protocol. This receiver uses a unidirectional serial connection to the aircraft to send commands for the throttle, yaw, pitch, and roll, and an extra command to change the flight mode. As the flight controller includes sensors and hardware tuned for this specific vehicle, we had to include a custom, lightweight, ESP32-CAM microcontroller, with onboard camera and Wi-Fi module, to substitute the Spektrum receiver (Figure 11). It meets all requirements and provides the opportunity to add more sensors and actuators if needed. To prevent delay issues by using a single device to capture camera images, manage the Wi-Fi connection and control the flight controller, the Spektrum messages simulator was put in a dedicated microcontroller, responsible for sending the command messages periodically. As such, even if the Wi-Fi connection or the camera add extra load in the ESP32, the other microcontroller is not affected and can react. For instance, it can detect a problem in the system and trigger a failsafe mechanism, such as an emergency landing.

**Figure 12:**
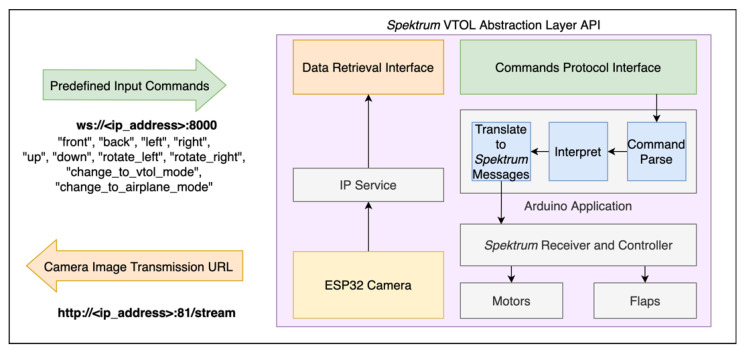
Spektrum based VTOL airplane abstraction layer, to provide access to the video camera transmission and flight control.

The abstraction layer created for this type of vehicles is represented in the Figure 12. As input, the vehicle waits for the control messages (e.g., front, back, change_to_vtol_mode). As output it provides the transmission of the video camera, through a predefined URL. As this vehicle is using the ESP32 camera, the video transmission is using an IP Service provided by an ESP32 library.

Whenever a message is received, the ESP32 parses the command, validates it, translates it to the corresponding Spektrum message and sends it via a Serial connection to the receiver. The control of the motors and flaps are ensured by the proprietary Spektrum receiver.

While interpreting the commands from the user, the ESP-32 also monitors the communication quality to detect eventual delays or interruptions in the connection. If the vehicle’s integrity becomes compromised, the safety system makes it land.

## 5. Solution Validation

After the prototyping phase, the validation of the solution occurs. Then, as the focus of this work is to create a scalable solution to provide users with services using UVs, the validation emphasizes the creation of multiple services, according to the vehicles integrated in the prototype. The successful integration of multiple vehicles and different services through the same platform may demonstrate the capacity of the solution to expand in users, services and vehicles up to a virtually infinite number.

**Figure 13:**
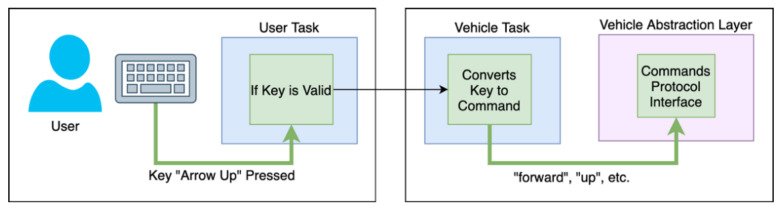
Representation of the control of a vehicle, through a computer keyboard.

For this purpose, we implemented three test scenarios, one for each vehicle type: car, drone, and VTOL airplane. The implemented services include the control and monitoring of each vehicle, in real-time, in which the users can freely guide the vehicles with a computer keyboard and, simultaneously, watch the UV’s camera images. The control procedure is described in Figure 13. The user task listens for the keyboard keys pressing, validates them and sends the input message to the vehicle task. Then, each key has a direct translation to a generic command that the abstraction layer of the vehicle is expecting. For the Internet communication, each vehicle was connected to a Wi-Fi router, and is identified by a public IP address. As multiple vehicles can be in the same network, they are distinguished by a unique port number.

### 5.1. Control and Monitoring of Multiple Custom-Built Cars

**Figure 14:**
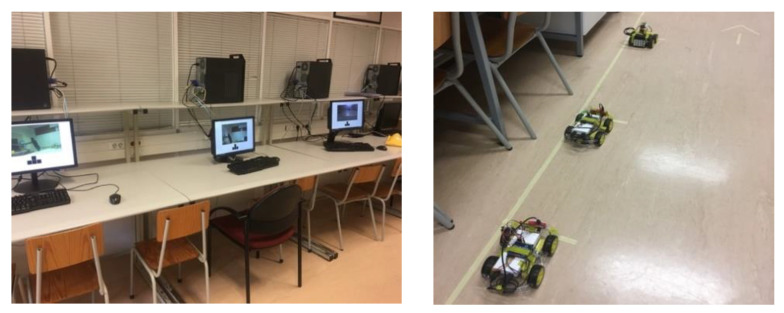
Control of multiple cars through the cloud platform with four computers.

We integrated four custom-built cars in the platform, available in the cloud via the Internet, to provide a manual control service. In three of the UGVs the camera was static, whilst in the remaining one it was mounted in a remotely controlled arm. Thus, the user tasks for these vehicles were adapted to include the only commands required to control the vehicle itself (Figure 14).

**Figure 15:**
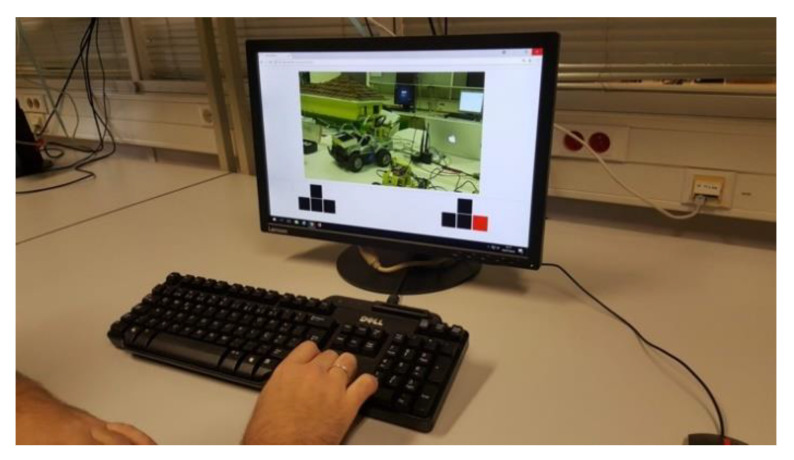
Control of the car with a controllable camera, which includes an extra input group to change its rotation.

In Figure 15 the control of the car equipped with a camera on a remotely controlled arm is represented, which relies on a more complex input scheme, divided in two groups made of four control buttons/keys each. In this case, the left group is used to move the camera up, down, left, or right, whilst the right one is used to control the vehicle forward, backward, leftward, or rightward.

Users were prompted to access the platform, select the control service and then pick one of the available cars, based on their characteristics (i.e., static or controllable camera). Meanwhile, other users could access the platform and request the service to spectate a specific vehicle, and therefore their user task was limited to watch the transmission of the video captured by the car’s camera.

In this test scenario it was possible to use the same platform and the same integrated vehicles to provide two different services, control and spectate, with user tasks adapted according to the vehicles’ specifications.

### 5.2. Control and Monitoring of a MAVLink Quadcopter

The control of a quadcopter, being an aerial vehicle, represents increased risks for people, the vehicle itself and the surrounding environment. Therefore, the control of the MAVLink compatible quadcopter was done step-by-step. First, we controlled it locally via a common GCS named QGroundControl. With this application, we were able to make the drone take-off, keep it stable at a 1.5 m height, and then land it safely. As everything was correctly assembled and running properly with the custom vehicle, we started the platform tests. In the platform, for the manual control of a drone, the users have three groups of buttons/keys. With the left group, users can make the vehicle go up, down, rotate to left and right. The right group provides inputs to move the vehicle forward, backward, leftward, and rightward. As emergency safety measures, the users task presents additional input options in the middle group to make the drone land immediately or return to the take-off site.

**Figure 16:**
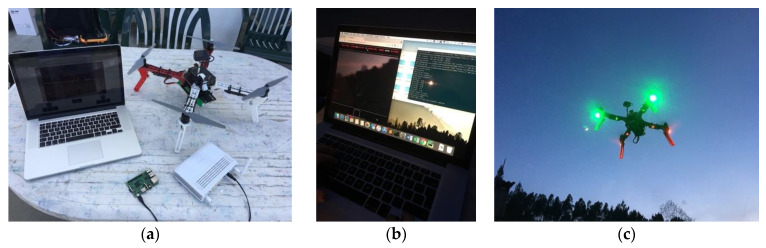
Representation of the control of a MAVLink quadcopter via the cloud platform: (**a**) presents the test scenario: a computer, a Wi-Fi router and the UAV; (**b**) the computer controls the quadcopter in real-time; (**c**) the quadcopter is manually controlled by a user, through the platform.

The test scenario is represented in Figure 16. In the first test with the platform, we replicated the test with the GCS. The vehicle took off successfully and remained stable at a 1.5 m height. Then, the land button was pressed, and it performed correctly. Afterwards, we tested each function. After the take-off, we assessed each single movement command. During these tests, we detected an issue with the local MAVProxy GCS. The left commands performed with success, whilst the right ones did not. For example, when issuing the command to go forward, the vehicle was going in an apparently random direction. Reviewing the implementation of the integration module and the MAVLink documentation, we figured out that the problem was in the “SET_POSITION_TARGET_LOCAL_NED” message. This message’s payload includes the speed in the X, Y and Z axes of the vehicle. These axes depend on a parameter called “coordinate_frame”. When users control drones, especially the commercial ones, they expect the speeds to be relative to the current vehicle heading direction. Although, in this implementation, the default value was expecting that the speed of each axis was relative to the magnetic north, using the cardinal directions as reference instead. Thus, after changing this option and defining the speeds according to its heading, the vehicle reacted as expected. This issue, once more, strengthens the potential of the platform to make the control of the vehicle transparent to its pilots and developers. With the proposed solution, users will not need to analyze the extensive MAVLink documentation, in this case, and fall in unnecessary problems when the goal is to control the vehicle in a standard way.

### 5.3. Control of a Spektrum VTOL Airplane

The VTOL airplane integrated in the platform was a commercial vehicle, and so it required external hardware, attached onboard, to connect to a local router and consequently to get an Internet connection. Fulfilling this requirement, it was integrated as the other two vehicle types, and made available in the platform for the two services of manual control and monitoring.

**Figure 17:**
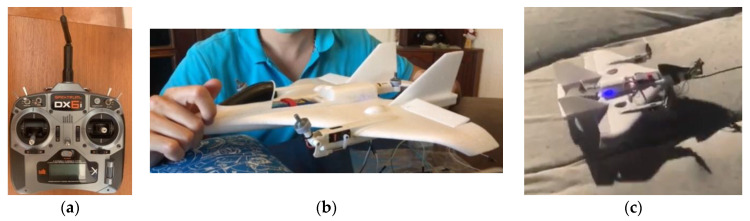
Representation of the Spektrum VTOL airplane: (**a**) Spektrum DX6i remote transmitter as the Spektrum VTOL controller; (**b**) the vehicle tests on bench; (**c**) control of the customized VTOL airplane through the platform.

The control mode of the VTOL airplane changes during its flight. First, in VTOL mode, it takes off vertically and flies as a tricopter [36]. During the flight, the user can change the disposition of the vehicle and make it act as an airplane, which is the aircraft mode. To understand better the control of this vehicle, we connected it with the recommended Spektrum transmitter made in United States of America (USA), Spektrum DX6i (Figure 17a), in the first tests. With this remote controller, we figured out the behavior of each stick. In VTOL mode, the left stick controls the motors’ throttle (up/down) and the yaw (left/right). The right stick controls the pitch (up/down) and the roll (left/right). In aircraft mode, the left stick controls the motors’ throttle (up/down) and the rudder (left/right), while the right stick controls the elevator (up/down) and the ailerons (left/right).

Considering the functionalities of this vehicle, during the control the user task of the control service provides three groups of keys/buttons. The left and right groups define the commands exactly as the correspondent sticks, while the middle group includes an option to change the flight mode from VTOL to aircraft and vice-versa. Thus, after piloting the vehicle with the traditional controller, the succeeding tests assessed the behavior of the UAV with each command function. For this purpose, and to ensure safety, we removed the propellers, and carefully secured the vehicle on a bench (Figure 17b). After testing all the functionalities, we finally controlled it via the platform and it reacted as expected (Figure 17c).

## 6. Tests, Results and Discussion

To evaluate the feasibility and performance of the communication under different conditions we created an assessment plan composed of three test cases, applied in three test scenarios, using four Raspberry Pi-based custom cars. Therefore, in this section, we present the testing phases, followed by the results and their discussion.

In Table 4 we present the test goals and the parameters used for the evaluation. The goal of the first test is to measure the average response time for the transmission and reception of a single message between the user and the vehicle, according to Equation (1). This value is particularly important to determine the sending interval between each message. Hence, in this test case we send 100 separate messages (i.e., 1000 ms interval). The average response time is used as the time interval for the following test cases. The test case number 2 intends to analyze the response time variations when the number of issued commands increase. At each step, we send multiple messages in a row, with the interval determined in test 1. The number of messages increases from 100, 250, 500, 750 to 1000. The goal of the third test case is to assess the control of multiple vehicles simultaneously. Then, we send messages to four different vehicles for 1, 5 and 10 min. The number of commands depends on the optimal average response time in each test scenario, according to Equation (2).

(1) $$\Delta_{responseTime}\;=\;transmissionTime+receptionTime$$

(2) $$numberOfCommands=\;\frac{controlTime}{averageResponseTime}$$

We performed the test cases in the scenarios presented in Table 5. The first test scenario corresponds to a local network, in a domestic environment. The next scenarios are distributed and based on the Internet. In the second, the devices communicate from remote networks, whilst in the last the devices communicate via 3G and LTE mobile networks. To evaluate the impact of the platform as gateway for the communications, we performed the tests in a point-to-point fashion and with the platform acting as gateway for all the messages between the users and the vehicles.

### 6.1. Local Network Scenario: Tests and Results

In this scenario, we connected the vehicles to a router via 2.4 GHz Wi-Fi, the user computer via 5 GHz Wi-Fi, to the same router, and hosted the platform in another computer with a 100 Mbps ethernet connection.

**Figure 18:**
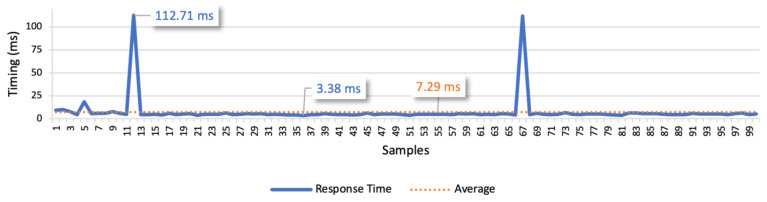
Results of the response times in a local network scenario, for the test case 1, in a point-to-point communication.

**Figure 19:**
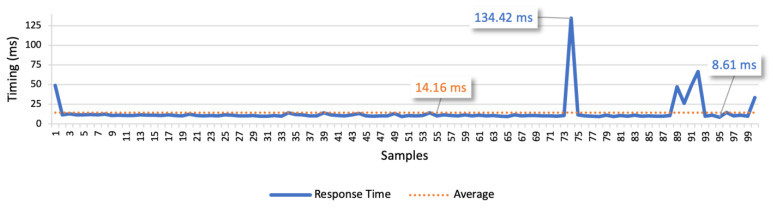
Results of the response times in a local network scenario, for the test case 1, communicating through the platform.

The results of test case 1 allow to find the response times for the isolated command messages. The point-to-point scenario is presented in Figure 18, whilst the scenario considering the communication through the platform is presented in Figure 19. These figures represent the minimum, the maximum and the average values. In the point-to-point communication, the minimum value is 3.38 ms, the maximum is 112.71 ms, and the average is 7.29 ms. We also considered the average deviation (4.53 ms) to find the optimal response time, which led us to 10 ms as the optimal time interval between commands. In the scenario considering the communication through the platform, the minimum value is 8.61 ms, the maximum is 134.42 ms, and the average is 14.16 ms. Considering the average deviation of 6.14 ms, we defined 20 ms as the optimal interval for the following tests.

The examination of the two figures allows to find a small number of outliers. These points may represent interferences from other devices in the network that cause an additional delay in some messages. Although, in most of the commands, the response time is near the average and stable. Comparing the average values of both the communication types, we can infer that the point-to-point approach is approximately two times faster.

In Table 6 we present the response times for the two different communication types, considering different numbers of commands, from 100 to 1000. For each test iteration, we calculated the minimum, the maximum, the average, and the average deviation response times. For the point-to-point communication, with a 10 ms interval, we got a minimum value of 2.45 ms, a maximum of 93.97 ms, the lower average of 3.61 ms with 0.86 ms average deviation, and the higher average of 5.81 ms with an average deviation of 2.84 ms. In the scenario considering the communication through the platform, with a 20 ms time interval, we got a minimum value of 3.63 ms, a maximum of 117.14 ms, the lower average of 5.54 ms with an average deviation of 1.95 ms, and the higher average of 6.82 ms with an average deviation of 3.48 ms.

The examination of the values for the different number of commands allowed to conclude that the minimum becomes lower when we send more commands. The difference between the point-to-point communication and the communication through the platform decreased, with a difference of approximately 1 ms. This small difference may be explained by the local network scenario and due to the optimization made in the first test case, finding the optimal response time.

The third test case intends to evaluate the solution in a real scenario, with four vehicles controlled at the same time. We present the obtained values in Table 7. For each communication type we controlled the vehicles for 1, 5 and 10 min. The number of commands is directly proportional to the duration of the tests, considering the predefined interval between each of them. As discussed in test case 1, this interval is different for each of the communication architectures (i.e., point-to-point or platform as middleware). Hence, we sent the following number of commands: for the point-to-point communication type, for 1 min we sent 6000 commands, for 5 min we sent 30,000 messages and for 10 min we sent 60,000 messages; for the communication through the platform, for 1 min we sent 3000 commands, for 5 min we sent 15,000 commands and for 10 min we sent 30,000 commands.

The examination of the obtained values allowed to conclude that the average response time for both communication types presents a difference of approximately 1 ms. The minimum values have a similar difference of approximately 1 ms. The maximum values in the platform approach are higher for 5 and 10 min, whilst in the 1-min case it was significantly lower. This lower value may be explained due to the fact that we send half the commands through the platform, when compared to the point-to-point communication type.

### 6.2. Remote Networks Scenario: Tests and Results

**Figure 20:**
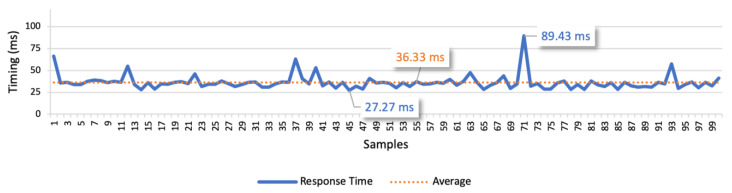
Results of the response times in the remote networks scenario, for the test case 1, in a point-to-point communication.

**Figure 21:**
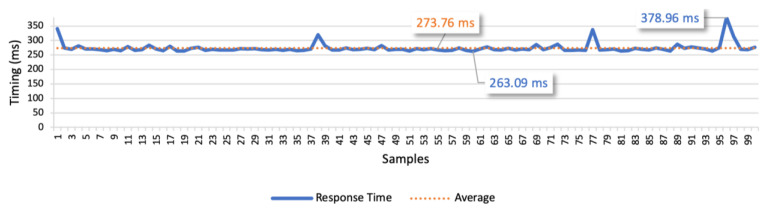
Results of the response times in the remote networks scenario, for the test case 1, communicating through the platform.

In this scenario the user and the vehicles are connected in different networks and communicate through the Internet via domestic fiber connections. The platform is hosted in an AWS EC2 instance, accessible via a public IP address. We performed the same three test cases as in the previous test scenario. The results of test case 1 allow to find the response times for the isolated command messages. The point-to-point scenario is presented in Figure 20, whilst the scenario considering the communication through the platform is presented in Figure 21. In the point-to-point communication, the minimum value is 27.27 ms, the maximum is 89.43 ms, and the average is 36.33 ms. We also considered the average deviation (4.58 ms) to find the optimal response time, which led us to 40 ms as the optimal time interval between commands. In the scenario considering the communication through the platform, the minimum value is 263.09 ms, the maximum is 378.96 ms, and the average is 273.76 ms. Considering the average deviation of 8.46 ms, we defined 280 ms as the optimal interval for the following tests.

The examination of the results allowed to find a slight variation in the response time in both communication approaches. Comparing the two figures, we can infer that the point-to-point approach is approximately seven times faster than the approach using the platform in the communication. This test demonstrates the high overhead of the platform when compared to a point-to-point communication.

We present in Table 8 the response times for the two different communication types and different numbers of commands, from 100 to 1000. For the point-to-point communication, with a 40 ms time interval, we got a minimum value of 23.30 ms, a maximum of 1061.54 ms, the lower average of 33.24 ms with 3.70 ms average deviation, and the higher average of 49.61 ms with an average deviation of 30.65 ms. In the scenario considering the communication through the platform, with a 280 ms time interval, we got a minimum value of 259.10 ms, a maximum of 1672.42 ms, the lower average of 269.51 ms with an average deviation of 5.46 ms, and the higher average of 558.96 ms with an average deviation of 408.84 ms.

The examination of the values allowed to conclude that the number of messages is not affecting the minimum response time significantly, whilst the maximum may present outliers in a higher number of messages. The difference between the point-to-point communication and the communication through the platform for the average response time is always greater than 200 ms. We conclude that the point-to-point connection presents, by far, the best results.

We present in Table 9 the results of the third test case. According to the time intervals specified in the test case 1, we sent the following number of commands: for the point-to-point communication type, for 1 min we sent 1500 commands, for 5 min we sent 7500 messages and for 10 min we sent 15,000 messages; for the communication through the platform, for 1 min we sent 214 commands, for 5 min we sent 1070 commands and for 10 min we sent 2140 commands.

The examination of the obtained values allowed to conclude that the average response time for both communication types is similar to the previous test case. Although, the analysis of the higher average deviation values, led us to conclude that the communication with the four vehicles through the platform was more prone to delays. This can be explained by the concurrence inside the platform, to distribute the packets to the multiple vehicles simultaneously. The maximum values are related to the establishment of the connection, that may result in longer responses for the first commands. As both communication types require the establishment of communication links, both present similar maximum values. As the point-to-point communication architecture presented better results, we extended the test to a period of 1 h, with an interval of 40 ms between commands, which corresponds to 90,000 commands. The results are presented in Table 10.

The analysis of the results allowed us to conclude that the minimum and maximum values are within a similar range, whilst the average and average deviation values increased approximately three times. These results can be justified by the number of issued commands, which is considerably higher, and the connection time, which is approximately six times longer than in the previous tests. Besides, an average value of 136 ms may be acceptable for most of the long-time services, as it represents seven or eight commands per second. With this test we can also conclude that, for long periods, it may be recommended to adjust the time interval to match the response time, which allows to optimize the balance between these two values.

### 6.3. Mobile Networks Scenario: Tests and Results

The difference in this scenario is in the communication between the user and the vehicles, which relies on 3G and LTE mobile networks. As we concluded in the previous scenarios that the point-to-point communication performed better, in this scenario we only considered that fashion. Moreover, as the presence of multiple vehicles did not affect the response times, we did not execute the test case 3 in this scenario.

**Figure 22:**
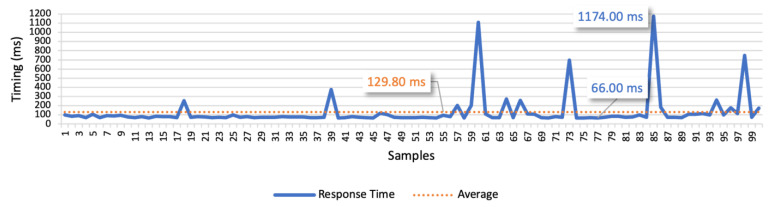
Results of the response times in a mobile network scenario (3G), for test case 1, in a point-to-point communication.

**Figure 23:**
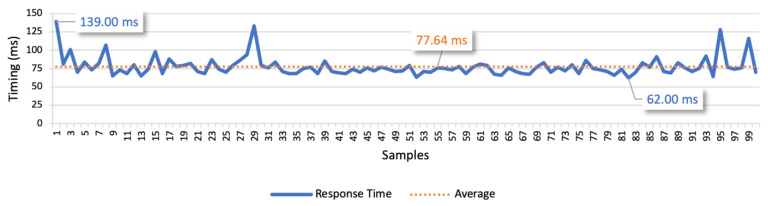
Results of the response times in a mobile network scenario (LTE), for test case 1, in a point-to-point communication.

The results of test case 1 allow to find the response times for the isolated command messages. The 3G connection scenario is presented in Figure 22, whilst Figure 23 presents the LTE connection scenario. In the 3G communication scenario, the minimum value is 66 ms, the maximum is 1174 ms, and the average is 129.80 ms. We also considered the average deviation (85.32 ms) to find the optimal response time, which led us to 160 ms as the optimal time interval between commands. In the LTE communication scenario, the minimum value is 62 ms, the maximum is 139 ms, and the average is 77.64 ms. Considering the average deviation of 8.46 ms, we defined 80 ms as the optimal interval for the following tests.

The examination of the 3G response values allowed to find a small number of outliers, whilst with LTE the range from the minimum and maximum values is much lower. The minimum values for both mobile networks are similar, but the average of LTE is approximately 1.5 times lower than the average of 3G. The visualization of the figures allowed us to conclude that the LTE network is more stable than the 3G, which presents more fluctuations in the response times.

We present in Table 11 the response times for the two different mobile network types and different numbers of commands, from 100 to 1000. For the 3G communication, with a 160 ms time interval, we got a minimum value of 58 ms, a maximum of 1720 ms, the lower average of 70.70 ms with 7.66 ms average deviation, and the higher average of 107.24 ms with an average deviation of 62.33 ms. On the LTE communication, with an 80 ms time interval, we got a minimum value of 49 ms, a maximum of 425 ms, the lower average of 65.91 ms with an average deviation of 6.96 ms, and the higher average of 81 ms with an average deviation of 19.25 ms.

The performance of mobile networks varies according to the day period [37]. As such, we also analyzed the peak and off-peak hours of mobile networks usage. According to [38], the rush hour corresponds to the 18:00 h–20:00 h day period, while the off-peak comes after 22:00 h. The peak hours usually correspond to the period when users leave their jobs and get more active on the Internet, whilst the off-peak hours correspond to inactive time when most people go to sleep. As such, we performed tests in both daytimes and present the results in Table 12. These tests only consider the LTE network, as it performed better in the previous tests and is more susceptible to overload and variable usage depending on the daytime period.

The values for the response time are higher during rush hours, and slightly decrease when we tested the prototype during the off-peak period. Whilst the minimum during the rush hour is lower than the off-peak period (47 ms against 48 ms), the average increased around 2 ms on the different test iterations. The maximum response time in the rush hour varied from 110 to 477 ms, whilst in the off-peak period the values resided in the 109–374 ms interval. Considering the overall differences, we can conclude that the rush hour period represents an overhead on the communications. Although, it is not considerably high and, consequently, critical for the control of the vehicles.

### 6.4. Discussion

The different test scenarios and cases allowed us to evaluate the solution under different conditions. In a local network scenario, the average response times reside in the 5–11 ms interval. Both in a point-to-point configuration or with the platform as the gateway, the values did not present considerable differences. In a distributed scenario, such as the one considering mobile networks, the values vary noticeable. Whilst in the point-to-point solution, the average values are in the 33–54 ms interval, when we introduced the platform as the middleware for the communications the average values increased to the 270–560 ms interval, depending on the presence of a single or many vehicles. We can conclude that the point-to-point solution is more efficient for the remote control of the vehicles. Considering this, we extended the test with the point-to-point architecture, to validate how the communication behaved for 60 min, a longer control interval. This extension was important to assess the variations of the response time in long-period services, and we concluded that it might be recommended to dynamically adjust the interval between messages to optimize the balance between the number of commands and the average response time.

We also tested the use of mobile networks (i.e., 3G and LTE) as communication technology. With 3G communication we got average response times of 70–108 ms, whilst with LTE communication the average response times decrease to 65–81 ms. The analysis of the average deviation time reveals high values when using the 3G communications, in opposition to the LTE communication. This means that the 3G link was more instable and presented high outliers during the emission and reception of multiple command messages. Considering the influence of daytime periods in the quality of service of mobile networks, we tested the performance of LTE during different hours, based on the on-peak (i.e., rush hours) and off-peak periods. These tests revealed a slight difference in the average response values. During rush hours, the average response time increased approximately 2 ms. Despite this difference, it is considerably low, and we expect it not to be critical for the control of the vehicles.

### 6.5. Recommendations

Following the discussion on tests, we conclude that recent technologies like fiber Internet and LTE potentiate real-time remote services. Besides, the new 5G communication technology promises lower latencies, considering values of up to 1 ms, so it may certainly improve the values obtained in the test cases. Although, the coverage of this mobile network technology worldwide it still limited, so it was not considered in the test scenarios.

The developers that intend to create new services on the top of this solution should keep the user abstracted from the details of the control service. In the past, users used to “sit in the driver’s seat”, which means that they were the ones controlling the services to reach a certain goal. Now, users should “sit in the passenger’s seat” and be driven to the goal without having special abilities or knowledge about how the services work.

The choice of correct and adaptable vehicles is a crucial step for the developers, as well. If the vehicle is already prepared for development, with SDKs or APIs, the creation of the abstract layer is easier. Besides, if the vehicle does not support development, it may require additional hardware. We went through this step to integrate the Spektrum VTOL airplane. The development effort for this vehicle was higher than on the other vehicles, as we needed to interpret the original vehicle logic and replay it.

Even though the platform is not a direct participant in the user–vehicle communication, it still monitors the services to ensure proper behaviors. The platform is also responsible to distribute the service tasks to the users and the vehicles. Whilst the task deployment does not affect the services, as it happens before the services start, multiple simultaneous requests to start services may cause congestions. To solve this specific issue, the AWS supports auto-scaling and auto-balancing, so the platform can be expanded and multiplied to distribute the handling of the requests.

## 7. Conclusions

Attending the benefits of UVs and following the trend to make services available through the Internet, in the cloud, we found an opportunity to combine both. According to the research we have done, the current solutions focus mainly on specific services and vehicles. Another limitation found in the researched work is related to the adopted communication architectures. In order to connect users and vehicles, the design of the protocols demands high technological knowledge.

Hence, we proposed a scalable and distributed architecture that is capable of providing a global access to multiple services, using different UV types. The cooperation between different UV types, communicating through the Internet, led us to define the Internet of Unmanned Vehicles (IoUV) concept. For this purpose, in our solution, each vehicle is generically prepared to be used in different services, through an abstraction layer with embedded safety features, transparent to users and developers, that works as an interface for its functionalities. Therefore, the implementation of each service is independent of the specificities of each vehicle type.

Following the solution proposal, we implemented a cloud-based prototype. Afterwards, we integrated three different vehicle types to accommodate three different services: small-sized cars, quadcopters, and VTOL airplanes. To evaluate the architecture scalability, we performed tests to measure the time from the moment that the user sends a command and receives its confirmation, under different scenarios and conditions. The communication between different remote networks performed well when the user communicated directly with the vehicle through a point-to-point connection, with an average of 36–54 ms. When we tested the platform to process and relay all the commands, the response times increased to 360–523 ms. Then, we can conclude that our approach of using a platform to distribute the services into multiple point-to-point connections is more efficient. Thus, this work answers to the research question, with the proposal of a scalable solution that provides UV-based services, accessible anywhere, at any time, for anyone.

As future work, asynchronous services should be implemented and tested. Along with that, more vehicle types and variations must be integrated to cover the different needs for future services, such as the example with the search and rescue activity. Finally, the platform should be made available for end-users to perform usability tests.

## Figures and Tables

**Table 1:** Comparison of different unmanned vehicles (UV) cloud platforms.

Ref.	Vehicle Types	Services	Development Scalability	Integration Complexity	Strengths	Weaknesses
[28]	UAV	Multiple	Via onboard (C++, Python) and remote (REST, WebSockets) APIs	High	Support for new services through a robust platform and APIs	The APIs require knowledge about MAVLinkOnly applicable to UAVs
[20]	UAV	Multiple	Via remote (Java–Rest, WebSockets) APIs	Medium	Users can connect directly to the UAV through different APIs	No embedded safety mechanismsOnly applicable to UAVs
[29]	UAV	Multiple	No	-	Robust service for search and rescue, through mission planning	No plans for development Only applicable to UAVs
[30]	UAV	Sensing	Via remote (REST) API	Medium	Point-to-point connections to the UAV through REST API	No embedded safety mechanismsOnly applicable to UAVs
[31]	UGV and UAV	Power lines inspection	No	-	Cooperation of UGV and UAV for power lines inspection	Plans for integrating new services and vehicles are not discussed
[32]	UGV	Surveillance and sensing	No	-	Robust architecture for Human to Machine interaction	Only applicable to UGVs and to that specific service
[33]	Robots and UAV	Search and rescue	No	-	Cooperation with robots and UAV for search and rescue	There is no possibility to integrate other services
This work	Multiple	Multiple	Deployment of user, platform and vehicle tasks	Low	Generic architecture for multiple UV types and services, through high-level APIs with embedded safety mechanisms	May require the development of abstraction layers for each vehicle type

**Table 2:** Attributes and respective meaning of the solution’s requirements.

Attribute	Meaning
Universal	Compatible with different devices
Diverse	Provide different services
Accessible	Any user should be able to use a service
Transparent	Users must only be aware of how to use the services
User-friendly	Services should be intuitive for the technologically illiterate users
Scalable	New services may be integrated, with low effort, without affecting the performance of the already integrated ones
Ubiquitous	Available from anywhere
Available	Available at anytime
Distributed	Distributed over different places for wide communication coverage

**Table 3:** Analysis of the MAVLink protocol, based on the controls that the proposed service tasks use.

Abstract. Layer Message	Description	MAVLink Message
arm, disarm	Change the system mode to armed/disarmed	MAV_CMD_DO_SET_MODE
take_off	Take-off from the ground, until a specified altitude	MAV_CMD_NAV_TAKEOFF
front, back, left, right, up, down	Move forward/backward, leftward/rightward and up/down	SET_POSITION_TARGET_LOCAL_NED
rotate_left, rotate_right	Rotate around its vertical axis, to the left/right	MAV_CMD_CONDITION_YAW
go_to_waypoint	Go to a specified location	MAV_CMD_NAV_WAYPOINT
land	Land the vehicle automatically	MAV_CMD_NAV_LAND
return_to_home	Move the vehicle back to the takeoff place, cruising at a predefined altitude	MAV_CMD_NAV_RETURN_TO_LAUNCH
battery_level, altitude, location, speed, flight_mode, is_armed, number_of_gps_satellites	Read the vehicle parameters, such as altitude or battery level	PARAM_REQUEST_READ

**Table 4:** Explanation of the goals, parameters and outputs of the three test cases.

Test Case	Test Goals	Test Parameters	Output
1	Find average response time for single messages	100 messages, with 1000 ms interval	Minimum, maximum, average, and average deviation response times
2	Analyze the evolution of the response time for multiple sequential messages	100, 250, 500, 750, and 1000 messages using as interval between messages the average response time obtained in test 1	
3	Assess the impact of controlling multiple vehicles simultaneously	Communicate with four vehicles, during 1, 5 and 10 min using as interval between messages the average response time obtained in test 1	

**Table 5:** Presentation of the three test scenarios and their test cases.

Test Scenario	Communication Type	Test Case 1	Test Case 2	Test Case 3
Local network	Point-to-point	✓	✓	✓
	Through the platform	✓	✓	✓
Remote networks	Point-to-point	✓	✓	✓
	Through the platform	✓	✓	✓
Mobile networks	Point-to-point	✓	✓	-

**Table 6:** Results of the test case 2 response times, in the local network scenario, to compare the different communication types under different loads.

Communication Type	Number of Commands	Minimum (ms)	Maximum (ms)	Average (ms)	Average Deviation (ms)
Point-to-point	100	3.53	39.62	5.14	2.44
	250	3.20	39.73	5.81	2.84
	500	3.15	27.38	4.80	1.22
	750	2.88	93.97	5.21	2.30
	1000	2.45	17.56	3.61	0.86
Through the platform	100	4.61	34.91	6.33	1.74
	250	4.11	117.14	6.82	3.48
	500	3.77	36.60	5.54	1.95
	750	3.74	37.69	6.40	2.80
	1000	3.63	98.80	6.62	3.99

**Table 7:** Results of the test case 3 response times, in the local network scenario, to compare the different communication types, with four vehicles.

Communication Type	Duration (m)	Minimum (ms)	Maximum (ms)	Average (ms)	Average Deviation (ms)
Point-to-point	1	2.69	463.23	6.30	3.21
	5	2.68	401.90	7.86	5.59
	10	2.64	406.53	7.23	4.59
Through the platform	1	3.71	219.49	7.53	3.03
	5	3.48	455.73	10.53	8.68
	10	3.61	536.59	8.24	4.18

**Table 8:** Results of the test case 2 response times, in the remote networks scenario, to compare the different communication types, under different loads.

Communication Type	Number of Commands	Minimum (ms)	Maximum (ms)	Average (ms)	Average Deviation (ms)
Point-to-point	100	25.51	108.82	36.61	6.51
	250	24.34	74.23	33.24	3.70
	500	23.88	1061.54	49.61	30.65
	750	23.46	168.38	34.69	5.10
	1000	23.30	141.65	34.52	4.93
Through the platform	100	259.50	375.33	269.51	5.46
	250	259.55	1320.55	303.13	63.66
	500	260.59	458.10	270.77	7.58
	750	259.10	398.36	270.14	6.32
	1000	259.76	1672.42	558.96	408.84

**Table 9:** Results of the test case 3 response times, in the remote networks scenario, to compare the different communication types, with four vehicles.

Communication Type	Duration (ms)	Minimum (ms)	Maximum (ms)	Average (ms)	Average Deviation (ms)
Point-to-point	1	24.95	1033.85	38.70	7.17
	5	23.60	524.05	36.51	6.45
	10	22.35	1231.65	54.00	37.63
Through the platform	1	258.62	391.53	271.08	6.22
	5	257.07	1403.14	523.07	376.17
	10	255.11	1867.02	368.53	176.66

**Table 10:** Results of the test used to assess an hour-long point-to-point communication in remote networks.

Communication Type	Duration (m)	Minimum (ms)	Maximum (ms)	Average (ms)	Average Deviation (ms)
Point-to-point	60	23.74	1360.76	136.03	113.89

**Table 11:** Results of the test case 2 response times, in the mobile networks scenario, to compare the different mobile network technologies, under different loads, in point-to-point communications.

Mobile Network Technologies	Number of Commands	Minimum (ms)	Maximum (ms)	Average (ms)	Average Deviation (ms)
3G	100	58.00	165.00	72.12	11.52
	250	60.00	904.00	107.24	62.33
	500	59.00	293.00	70.70	7.66
	750	60.00	606.00	78.68	17.46
	1000	58.00	1720.00	89.34	36.82
LTE	100	49.00	99.00	65.91	6.96
	250	51.00	111.00	69.94	7.54
	500	49.00	154.00	67.82	7.20
	750	49.00	216.00	67.25	6.64
	1000	45.00	425.00	81.00	19.25

**Table 12:** Results of the response times, in the LTE mobile network scenario, in different daytime conditions (i.e., rush hour and off-peak period).

Daytime Condition	Number of Commands	Minimum (ms)	Maximum (ms)	Average (ms)	Average Deviation (ms)
Rush hour	100	56.00	110.00	71.81	6.17
	250	56.00	140.00	72.12	6.84
	500	54.00	166.00	71.63	6.67
	750	47.00	297.00	71.69	8.12
	1000	47.00	477.00	71.97	10.34
Off-peak period	100	53.00	109.00	69.39	5.39
	250	51.00	158.00	69.70	8.58
	500	50.00	374.00	70.78	12.05
	750	51.00	165.00	70.81	6.90
	1000	48.00	156.00	69.34	5.97

## Data Availability

Not applicable.
